# Supplementary material for: Using machine learning and big data to explore the drug resistance landscape in HIV
Source: PLoS Comput Biol. 2021 Aug 26;17(8):e1008873. doi: 10.1371/journal.pcbi.1008873 (PMC8425536; doi:10.1371/journal.pcbi.1008873)
Supplement: S1 Appendix — Technical details about the implementation and options for used tools. (PDF) [file pcbi.1008873.s001.pdf]

S1 Appendix. Technical appendix

Data

Data Availability

The policy of the UK HIV Drug Resistance Database is to make DNA sequences available to any bona fide researcher who submits a scientifically robust proposal, provided data exchange complies with Information Governance and Data Security Policies in all the relevant countries. This includes replication of findings from published studies, although the researcher would be encouraged to work with the main author of the published paper to understand the nuances of the data. Enquiries should be addressed to iph.hivrdb@ucl.ac.uk in the first instance. More information on the UK dataset is also available on the UK CHIC homepage: www.ukchic.org.uk. Amino acid sequences are made available along with a metadata file.

The West and central African dataset is available as supplementary information along with a metadata file containing HIV subtype, treatment information and known RAM presence/absence for each sequence.

Predictions made for each sequence of both datasets, by all of the trained classifiers are made available as part of the supplementary data as well as synthetic results from which the figures of the paper were drawn. The importance values for each mutation and each trained classifier are also made available.

All the data and metadata files made available are hosted in the online repository linked to this project at the following URL:
github.com/lucblassel/HIV-DRM-machine-learning/tree/main/data

Data Preprocessing

For both the African and UK datasets, the sequences were truncated to keep sites 41 to 235 of the RT protein sequence before encoding. This truncation was needed to avoid the perturbation to classifier training due to long gappy regions at the beginning and end of the UK RT alignment caused by shorter sequences. These positions were determined with the Gblocks software [3] with default parameters, except for the Maximum number of sequences for a flanking position, set to

50,000, and the Allowed gap positions, which was set to "All". The encoding was done with the `OneHotEncoder` from the category-encoders python module [4].

Classifiers

We used classifier implementations from the scikit-learn python library [5], `RandomForestClassifier` for the random forest classifier, `MultinomialNB` for Naïve Bayes and `LogisticRegressionCV` for logistic regression.

`RandomForestClassifier` was used with default parameters except:

- `"n_jobs"=4`
- `"n_estimators"=5000`

`LogisticRegressionCV` was used with the following parameters:

- `"n_jobs"=4`
- `"cv"=10`
- `"Cs"=100`
- `"penalty"='l1'`
- `"multi_class"='multinomial'`
- `"solver"='saga'`
- `"scoring"='balanced_accuracy'`

`MultinomialNB` was used with default parameters.

For the Fisher exact tests, we used the implementation from the scipy python library [6], and corrected p-values for multiple testing with the statsmodels python library [7] using the `"Bonferroni"` method.

Scoring

To evaluate classifier performance several measures were used. We computed balanced accuracy instead of classical accuracy, because it can be overly optimistic, especially when assessing a highly biased classifier on an unbalanced test set [1].The balanced accuracy is computed using the following

formula, where TP and TN are the number of true positives and true negatives respectively, and FP and FN are the number of false positives and false negatives respectively:

$$balanced\ accuracy = \frac{1}{2}\left(\frac{TP}{TP+FP} + \frac{TN}{TN+FN}\right)$$

We also computed adjusted mutual information (AMI). We chose it over mutual information (MI) because it has an upper bound of 1 for a perfect classifier and is not dependent on the size of the test set, allowing us to compare the performance for differently sized test sets [2]. The adjusted mutual information of variables U and V is defined by the following formula, where $MI(U,V)$ is the mutual information between variables U and V, $H(X)$ is the entropy of the variable X ($= U$ or V) and $E\{MI(U,V)\}$ is the expected MI, as explained in [8].

$$AMI(U,V) = \frac{MI(U,V) - E\{MI(U,V)\}}{\frac{1}{2}[H(U)+H(V)] - E\{MI(U,V)\}}$$

MI was used to compute the G statistic, which follows the chi-square distribution under the null hypothesis [9]. This was used to compute p-values for each of our classifiers and assess the significance of their performance. G is defined by equation below, where N is the number of samples.

$$G = 2 \cdot N \cdot MI(U,V)$$

Finally, to check the probabilistic predictive power of the classifiers we also computed the Brier score which is the mean squared difference between the ground truth and the predicted probability of being of the positive class for every sequence in the test set (therefore lower is better for this metric). The Brier score is defined in equation below, where p_t is the predicted probability of being of the positive class for sample t and o_t is the actual class (0 or 1, 1=positive class) of sample t:

$$Brier\ score = \frac{1}{N}\sum_{t=1}^{N}(p_t - o_t)^2$$

We used the following implementations from the scikit-learn python library [5] with default options:

- `balanced_accuracy_score`
- `mutual_info_score`
- `adjusted_mutual_info_score`
- `brier_score_loss`

We used the relative risk to observe the relationship between one of our new mutations and a binary character X such as treatment status or presence/absence of a known RAM.

$$RR(new, X) = \frac{prevalence\,(new\ mutation \mid X = 1)}{prevalence\,(new\ mutation \mid X = 0)}$$

$$= \frac{|(new = 1) \cap (X = 1)|}{|(X = 1)|} \div \frac{|(new = 1) \cap (X = 0)|}{|(X = 0)|}$$

References

1. Brodersen KH, Ong CS, Stephan KE, Buhmann JM. The Balanced Accuracy and Its Posterior Distribution. In: 2010 20th International Conference on Pattern Recognition; 2010. p. 3121–3124.

2. Vinh NX, Epps J, Bailey J. Information Theoretic Measures for Clusterings Comparison: Variants, Properties, Normalization and Correction for Chance. Journal of Machine Learning Research. 2010;11:18.

3. Castresana J. Selection of Conserved Blocks from Multiple Alignments for Their Use in Phylogenetic Analysis. Molecular Biology and Evolution. 2000;17(4):540–552. doi:10.1093/oxfordjournals.molbev.a026334.

4. McGinnis W, Hbghhy, Tao W, Andrethrill, Siu C, Davison C, et al.. Scikit-Learn-Contrib/Categorical-Encoding: Release For Zenodo; 2018. Zenodo. doi:10.5281/zenodo.1157110

5. Pedregosa F, Varoquaux G, Gramfort A, Michel V, Thirion B, Grisel O, et al. Scikit-Learn: Machine Learning in Python. Journal of Machine Learning Research. 2011;12(Oct):2825–2830.

6. Virtanen P, Gommers R, Oliphant TE, Haberland M, Reddy T, Cournapeau D, et al. SciPy 1.0: Fundamental Algorithms for Scientific Computing in Python. Nature Methods. 2020;17(3):261–272. doi:10.1038/s41592-019-0686-2.

7. Skipper Seabold, Josef Perktold. Statsmodels: Econometric and Statistical Modeling with Python. In: Stéfan van der Walt, Jarrod Millman, editors. Proceedings of the 9th Python in Science Conference; 2010. p. 92 – 96.

8. Vinh NX, Epps J. A Novel Approach for Automatic Number of Clusters Detection in Microarray Data Based on Consensus Clustering. In: 2009 Ninth IEEE International Conference on Bioinformatics and BioEngineering; 2009. p. 84–91.

9. Harremoes P. Mutual Information of Contingency Tables and Related Inequalities. In: 2014 IEEE International Symposium on Information Theory. Honolulu, HI, USA: IEEE; 2014. p. 2474–2478.
