# Supplementary material for: Using machine learning and big data to explore the drug resistance landscape in HIV
Source: PLoS Comput Biol. 2021 Aug 26;17(8):e1008873. doi: 10.1371/journal.pcbi.1008873 (PMC8425536; doi:10.1371/journal.pcbi.1008873)
Supplement: S2 Appendix — A detailed explanation of the procedure followed to test pairs of mutations for association with treatment. Detailed numerical results are also given. (PDF) [file pcbi.1008873.s002.pdf]

S6 Appendix. Fisher exact tests on pairs of mutations

In order to study epistasis further we conducted conducted Fisher exact tests between every pair of mutations in the UK dataset ($n = 867,903$) and the treatment status, corrected the p-values with the Bonferroni method with an overall risk level $\alpha = 0.05$.

Out of these tests, $1,309$ pairs were significantly associated with treatment status. 424 out of $1,309$ these pairs were two known RAMs, 806 of these pairs contained one known RAM and only 79 tests had pairs involving no known RAM at all. Furthermore out of these $1,309$ significantly associated pairs, 829 contained two mutations that were significantly associated to treatment when testing mutations one by one. In 478 pairs, one of the two mutations is associated to treatment on its own, and the remaining 2 pairs, none of the mutations were significantly associated with treatment on their own.

These 2 pairs were K103R + V179D and T165I + K173Q. The first pair, is a pair of known RAMs and this interaction is characterized in the HIVDb database (https://hivdb.stanford.edu/dr-summary/comments/NNRTI/). The second pair is made up of new mutations, and the corrected p-value is 0.02. In the Standford HIVDB, T165I has been associated to a reduction in EFV susceptibility.

Out of the $1,309$ pairs significantly associated to treatment, 151 contained at least one of our 6 new potential RAMs, in 6 cases the pair was made up of 2 of them.

In the UK dataset, phylogenetic correlation is likely very impactful with regards to these tests. Indeed, the sequences are far from being independent. In order to alleviate this effect we decided to test the sigficative pairs again on the African dataset, and once more correct with the Bonferroni procedure.

Out of the $1,309$ tests 294 have significative p-values after correction. Out of these 221 pairs were composed of 2 mutations individually significatively associated with treatment. The remaining 73 pairs had one mutation significantly associated with treatment.

Out of the 221 significative tests, 156 pairs were composed of 2 known RAMS while 135 had one known RAM in the pair. The remaining 3 pairs that do not contain a known RAM all contained either L228R or L228H which are both part of our 6 potential RAMS.
