# Supplementary figures and images for: Using machine learning and big data to explore the drug resistance landscape in HIV
Source: PLoS Comput Biol. 2021 Aug 26;17(8):e1008873. doi: 10.1371/journal.pcbi.1008873 (PMC8425536; doi:10.1371/journal.pcbi.1008873)

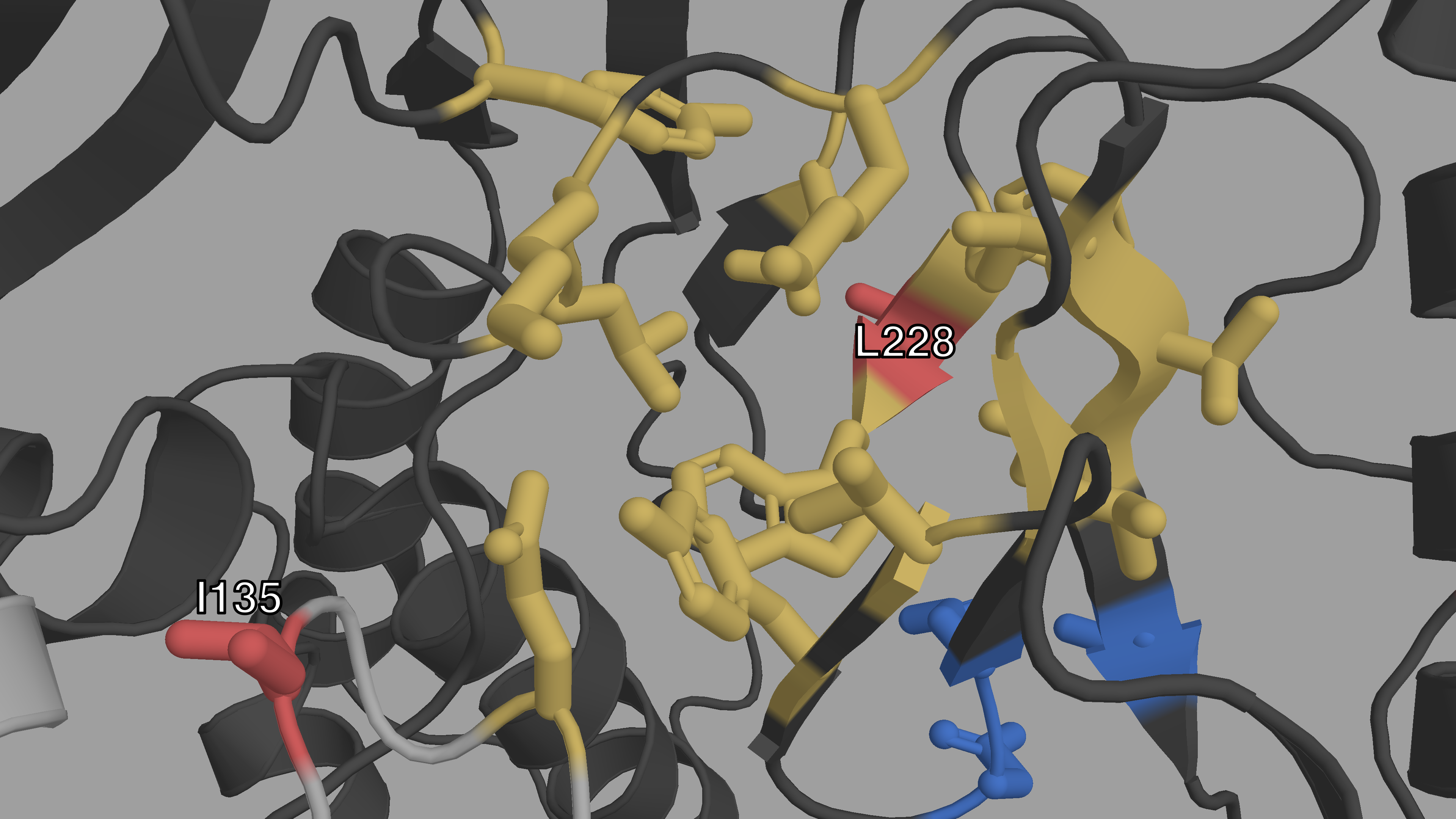

Supplement: S2 Fig — The p66 subunit is colored in dark gray, the p51 subunit in light gray. The NNIBP is highlighted in yellow. The active site is colored in blue. We can see the physical proximity of I135 (red) to the entrance of the NNIBP. We can also see how L228 (red) is between 2 AAs of the NNIBP. (PNG) [file pcbi.1008873.s004.png]

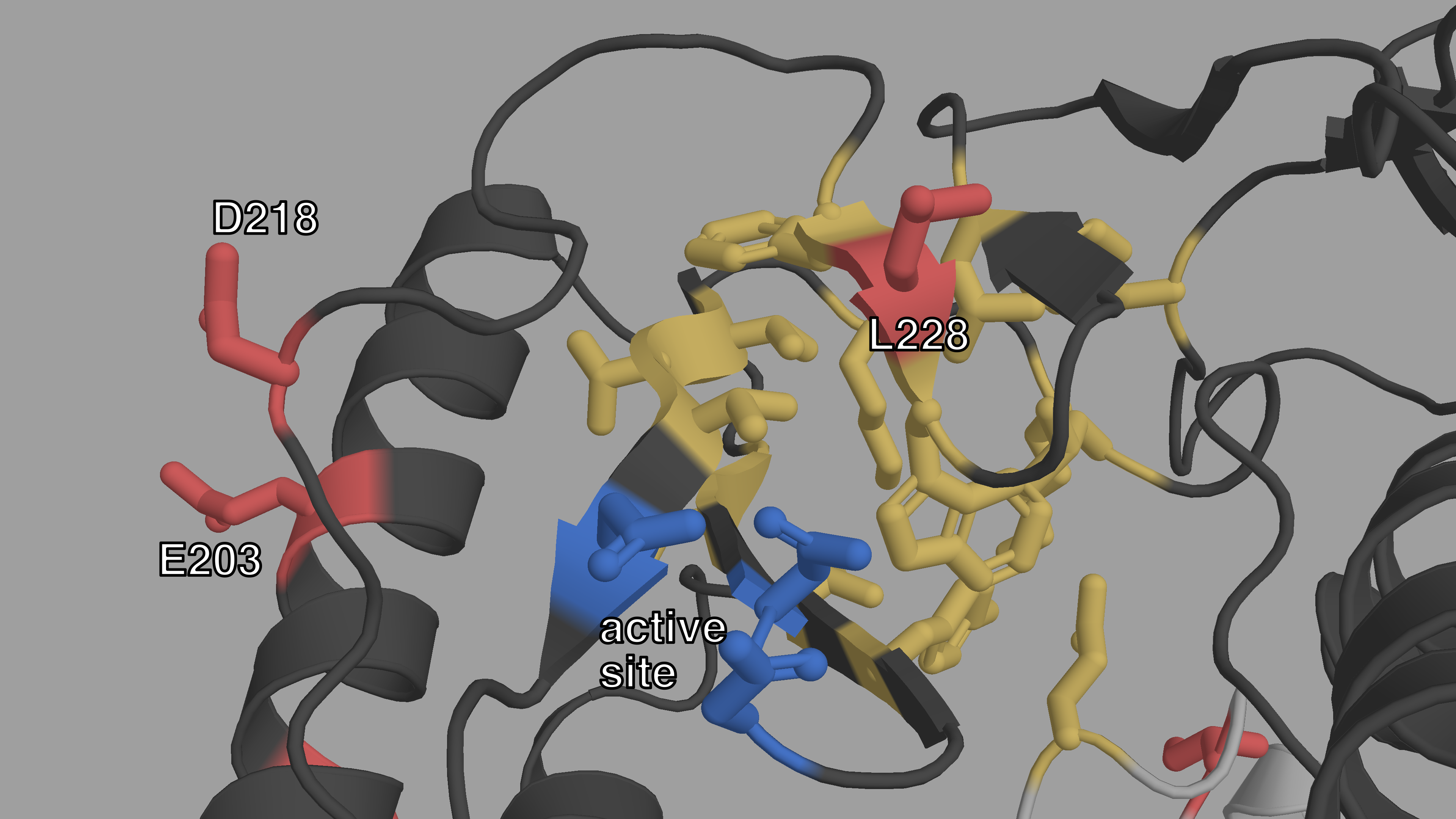

Supplement: S3 Fig — The p66 subunit is colored in dark gray, the p51 subunit in light gray. The active site is highlighted in blue. The NNIBP is colored in yellow. L228, E203 and D218 (red) are also very close on either side of the active site. (PNG) [file pcbi.1008873.s005.png]
