# Supplementary material for: Using machine learning and big data to explore the drug resistance landscape in HIV
Source: PLoS Comput Biol. 2021 Aug 26;17(8):e1008873. doi: 10.1371/journal.pcbi.1008873 (PMC8425536; doi:10.1371/journal.pcbi.1008873)
Supplement: S1 Table — (PDF) [file pcbi.1008873.s006.pdf]

S5 Table. Detailed view of the characteristics of new potential RAMs

	rank		codon distance			UK			Africa					Dayhoff	Change in			
	T/N	W/W	min	UK	Africa	count	ratio $\rho(new, treatment)$	$\rho(new, with\ RAM)$	count	ratio $\rho(new, treatment)$	$\rho(new, with\ RAM)$	p-value	B62	category shift	net charge	polarity	hydrophobicity index	molecular weight
**L228R**	0	0	1	1.16	1.21	227 (0.4%)	18.1 [12.9;27.3]	115.7 [55.1;507.3]	98 (2.5%)	32.5[15.4;147.1]	42.4 [17.8;∞]	2.0E-30	-2	e → d	1	5.6	-0.93	43.03
**E203K**	1	1	1	1.31	1.33	256 (0.5%)	11.0 [8.2;15.1]	20.1 [13.7;32.1]	56 (1.4%)	14.1[6.7;71.9]	17.4 [8.2;83.7]	6.4E-14	1	c → d	2	-1	0.68	-0.94
**D218E**	2	3	1	1	1	168 (0.3%)	13.1 [9.0;19.6]	27.0 [16.3;57.0]	25 (0.6%)	∞ [∞;∞]	∞ [∞;∞]	2.0E-09	2	c → c	0	-0.7	0.01	14.03
**L228H**	3	4	1	1.12	1.17	287 (0.5%)	6.4 [5.1;8.4]	9.2 [6.9;12.6]	53 (1.3%)	23.1[9.4;∞]	34.1 [12.0;∞]	2.7E-15	-3	e → d	0	5.5	-0.92	23.99
**I135L**	4	6	1	1.16	1.13	540 (1.0%)	1.8 [1.5;2.1]	2.4 [2.0;2.8]	134(3.4%)	2.6 [1.8;3.8]	2.4 [1.7;3.4]	2.6E-07	2	e → e	0	-0.3	-0.69	0
**H208Y**	8	9	1	1.10	1.12	205 (0.4%)	8.8 [6.5;12.5]	14.9 [9.9;23.6]	13 (0.3%)	∞ [∞;∞]	∞ [∞;∞]	7.3E-05	2	d → f	0	-4.2	1.27	26.03

Rank: For each new mutation we computed the aggregate feature importance ranks for the RTI-naive / RTI-experienced and known RAM present / known RAM absent classification tasks. **Codon distance:** We computed the minimum number of nucleotide mutations to go from the wild amino acid codons to those of the mutated amino acid, as well as the average codon distance between both amino acids, weighted by the prevalence of each wild and mutated codon in the UK and the African datasets. **Count (both UK and Africa):** We looked at the number of apparitions of each new potential RAM in the UK and African datasets and the corresponding prevalence in parentheses. **Ratio (both UK and Africa):** We computed the prevalence ratio $\rho(new, treatment)$ (e.g. L228R is 18.1 times more prevalent in RTI-experienced sequences compared to RTI-naive sequences in the UK dataset). We also computed the prevalence ratio $\rho(new, anyRAM)$ (e.g. L228R is 115.7 times more prevalent in sequences that have at least one known RAM than in sequences that have none in the UK dataset). The 95% confidence intervals shown under each ratio were computed with 1000 bootstrap samples of size $n = 55,000$ drawn with replacement from the whole UK dataset (The same procedure was done on the African dataset with size $n = 3990$). **p-values:** Fisher exact tests were done on the African dataset to see if each of these new mutations were more prevalent in RTI-experienced sequences; p-value were corrected with the Bonferroni method for the six simultaneous tests. **B62:** BLOSUM62 similarity values (e.g. D218E = 2, reflecting that E and D are both negatively charged and highly similar). **Dayhoff category shift:** The change in Dayhoff amino acid category is written thusly: "starting category → ending category". These categories are as follows: *a:* Sulfur polymerization. *b:* Small, *c:* Acid and amide, *d:* Basic, *e:* Hydrophobic and *f:* aromatic. **Physico-chemical change:** Change in physicochemical properties was obtained by subtracting the property value of the wil-type amino acid from the mutated amino acid. All values were obtained from the AAindex database (Kawashima S, Pokarowski P, Pokarowska M, Kolinski A, Katayama T, Kanehisa M. AAindex: amino acid index database, progress report 2008. Nucleic acids research. 2007 Nov 12;36(suppl_1):D202-5.)
